# Complete chloroplast genome of the medicinal plant *Amomum compactum*: gene organization, comparative analysis, and phylogenetic relationships within Zingiberales

**DOI:** 10.1186/s13020-018-0164-2

**Published:** 2018-02-13

**Authors:** Ming-li Wu, Qing Li, Jiang Xu, Xi-wen Li

**Affiliations:** 1 Institute of Chinese Materia Medica, China Academy of Chinese Medical Sciences, Dongcheng District, Dongzhimen Nanxiaojie within 16, Beijing, 100700 China; 2 Pharmacy Faculty, Hubei University of Chinese Medicine, No. 1, Huangjiahu West Road, Hongshan District, Wuhan, 430065 Hubei China; 3 Department of Pharmacy, Changzheng Hospital, Second Military Medical University, No. 415 Fengyang Road, Huangpu District, Shanghai, 200003 China

**Keywords:** *Amomum compactum*, Chloroplast genome, SSR, Phylogeny, High-throughput sequencing technology

## Abstract

**Background:**

*Amomum compactum* is one of the basic species of the traditional herbal medicine amomi fructus rotundus, with great pharmacology effect. The system position of *A. compactum* is not clear yet, and the introduction of this plant has been hindered by many plant diseases. However, the correlational molecular studies are relatively scarce.

**Methods:**

The total chloroplast (cp) DNA was extracted according to previous studies, and then sequenced by 454 GS FLX Titanium platform. Sequence assembly was complished by Newbler. Genome annotation was preformed by CPGAVAS and tRNA-SCAN. Then, general characteristics of the *A. compactum* cp genome and genome comparsion with three Zingiberaceae species was analyzed by corresponding softwares. Additionally, phylogenetical trees were reconstructed, based on the shared protein-coding gene sequences among 15 plant taxa by maximum parsimony (MP) and maximum likelihood (ML) methods.

**Results:**

The *A. compactum* cp genome with a classic quadripartite structure, consisting of a pair of reverse complement repeat regions (IRa/IRb) of 29,824 bp, a large single copy (LSC, 88,535 bp) region as well as a small single copy (SSC, 15,370 bp) region, is 163,553 bp in total size. The total GC content of this cp genome is 36.0%. The *A. compactum* cp genome owns 135 functional genes, that 113 genes are unique, containing eighty protein-coding genes, twenty-nine tRNA (transfer RNA) genes and four rRNA (ribosomal RNA) genes. Codon usage of the *A. compactum* cp genome is biased toward codons ending with A/T. Total 58 SSR loci and 24 large repeats are detected in the *A. compactum* cp genome. Relative to three other Zingiberaceae cp genomes, the *A. compactum* cp genome exhibits an obvious expansion in the IR regions. In *A. compactum* cp genome, the *ycf1* pseudogene is 2969 bp away from the IRa/SSC border, whereas in other Zingiberaceae species, it is only 4–5 bp away from the IRa/SSC border. Comparative cp genome sequences analysis of *A. compactum* with other Zingiberaceae reveals that the gene order and gene content differ slightly among Zingiberaceae species. The phylogenetic analysis based on 67 protein-coding gene sequences supports the phylogenetic position of *A. compactum*.

**Conclusions:**

The study has identified unique features of the *A. compactum* cp genome which would be helpful for us to understand the cp genome evolution and offer useful information for phylogenetics and further studies of this traditional medicinal plant.

**Electronic supplementary material:**

The online version of this article (10.1186/s13020-018-0164-2) contains supplementary material, which is available to authorized users.

## Background

Chloroplasts can provide necessary energy for plants growth as photosynthetic organelles, which also participate in other major life activities such as starch storage, sugar synthesis and many critical biological metabolic pathways. As circular DNA molecules, cp genomes mainly vary from 120 to 160 kb in size with a typical quadripartite organization in angiosperms [1]. Two reverse complement copies of IR region (20–28 kb) separate the whole cp genome into a LSC region (80–90 kb) and a SSC region (16–27 kb) [2]. In angiosperms, cp genomes usually encode approximately 80 unique proteins, 30 tRNAs and four rRNAs. Previous studies have corroborated that cp gene order, gene content, and genome organization are highly conserved in plants [3, 4]. Owing to the high conservation and monolepsis, cp genomes are widely used in species identification, phyletic evolution studies and genetic engineering. The availability of whole cp genomes has helped to resolve phylogenetic relationships among major clades of angiosperms with greater accuracy [5, 6]. Nevertheless, with the number of cp genomes increasing, gene losses, structural rearrangements and IR contractions/expansions have been reported, which can also be exploited for the reconstruction of plant phylogenies [7–9].

*Amomum compactum* (genus *Amomum*, family Zingiberaceae) is one of the basic species of the traditional Chinese medicine *amomi fructus rotundus*, which is mainly produced in Vietnam and Thailand and is cultivated as a medicinal plant in the Guangdong, Guangxi and Yunnan provinces of China with great pharmacology effect. However, bacterial wilt, damping-off, leaf spot and other major plant diseases have become a severe obstacle for the introduction of this plant. Many plants belonging to the Zingiberaceae family are used as important seasoning and medicinal plants, such as *Zingiber officinale*, *Amomum villosum*, *Curcuma longa*, *Zingiber mioga*, *Elettaria cardamomum*, and *Alpinia officinarum*. In addition, previous studies have shown that the efficacy, chemical composition and pharmacological effects among the five genera of Zingiberaceae are strongly correlated. It is of great significance and broad interest to investigate the genetic relationships of traditional Chinese medicinal plants to find alternative medicinal plants. With the number of whole cp genomes in the Zingiberaceae increasing, the cp genome sequences of other species in Zingiberaceae are becoming easier to be assembled. However, studies of amomi fructus rotundus are scarce both inside and outside China, especially molecular studies.

This study reports the assembly, annotation and structural analysis of *A. compactum* cp genome for the first time. And to reveal the structure of this cp genome, we compare the organization (IR expansion/contraction and divergent regions) of complete cp genomes between *A. compactum* and other Zingiberaceae species. We also provide the result of phylogenetic analyses on basis of 67 protein-coding gene sequences from *A. compactum* and 14 monocot cp genomes.

## Methods

### DNA extraction and sequencing

Fresh *A. compactum* leaves were acquired from cultivated bases in Guangdong Province, China. The total cp DNA was extracted from roughly 100 g of leaves through an improved method by Li et al. [10]. The quality of cp DNA was checked by Nanodrop-2000 spectrometer (Nanodrop Technologies, Wilmington, DE, USA), and agarose gel electrophoresis. Pure cp DNA was used for shotgun library construction with 454 GS FLX Titanium platform. The obtained SFF file was preprocessed by trimming short (L < 50 bp) and low-quality (Q < 20) reads. Trimmed reads were assembled using Newbler V2.6 (GS FLX De Novo Assembler Software). In order to verify the assembly, the four junctional regions were further confirmed by Sanger sequencing.

### Genome assembly and annotation

Preliminary gene annotation of this cp genome was performed by CpGAVAS, a program available online (http://www.herbalgenomics.org/0506/cpgavas) [11]. The position of each gene was then manually corrected by Apollo [12] after alignment to the reference genomes by MEGA 5.0. In addition, according to start and stop codons, minor revisions were performed. The tRNAs were further confirmed by the online tool tRNAscan-SE with default settings (http://lowelab.ucsc.edu/tRNAscan-SE/). [13]. Then, the circular map of this cp genome was accomplished by OrganellarGenomeDRAW program (http://ogdraw.mpimp-golm.mpg.de/) [14]. Finally, the complete cp genome of *A. compactum* was submitted to NCBI GenBank database (Accession Number: MG000589).

### Sequence analyses

Relative synonymous codon usage (RSCU) values, which were used to research the features of variations in synonymous and nonsynonymous codon usage by disregarding the composition impact of amino acid, were determined using MEGA 6.0 [15]. Additionally, GC content and codon usage were determined by MEGA 6.0. SSRs (simple sequence repeats) loci were detected by MISA software (http://pgrc.ipk-gatersleben.de/misa/), with following thresholds: ten, six, five, five, five, and five repeat units for mono-nucleotide, di-nucleotide, tri-nucleotide, tetra-nucleotide, penta-nucleotide, and hexa-nucleotide SSRs, respectively. To analyze the repeat structure, REPuter [16] (http://bibiserv.techfak.uni-bielefeld.de/reputer/) was performed to detect forward (direct) and palindromic (inverted) repeats in the cp genome. The minimum repeat unit was set to 30 bp in length, the identity of repeats was set to > 90%, and the Hamming distance equals three. All identified results were verified and redundant repeats were manually removed.

### Genome comparison

Pairwise alignments of several cp genome sequences were conducted by MUMmer [17], and the dot plots were drawn using a Perl script. The complete cp genomes of *A. compactum* and three other Zingiberaceae species (Additional file 1), *Curcuma flaviflora* (KR967361), *Curcuma roscoeana* (KF601574), and *Zingiber spectabile* (JX088661), were used for comparative analysis by mVISTA program (http://genome.lbl.gov/vista/index.shtml) [18] in Shuffle-LAGAN mode. *A. compactum* was set as the reference.

### Phylogenomic analysis

To examine the phylogenetic position of *A. compactum*, 14 complete chloroplast genomes were downloaded from NCBI. The 67 shared protein-coding gene sequences were extracted using a Python script and aligned separately by ClustalW2. Phylogenetical trees were reconstructed based on 67 concatenated protein-coding gene sequences by MP and ML methods. The best-fitting model was filtrated by jModelTest 2.1.7 through the Akaike information criterion (AIC) [19]. The MP tree was reconstructed by PAUP ver. 4.0b10 [20] with a heuristic search, while ML analysis was calculated by RAxML-HPC 2.7.6.3 on XSEDE in the CIPRES Science Gateway (http://www.phylo.org/) with default parameters. Based on APGIII, *Fritillaria cirrhosa* was set as an outgroup. Both MP and ML analyses used 1000 bootstrap replicates.

The Minimum Standards of Reporting Checklist includes details of the experimental design, statistics, and resources used in this study.

## Results and discussion

### General characteristics of the *A. compactum* cp genome

**Fig. 1:**
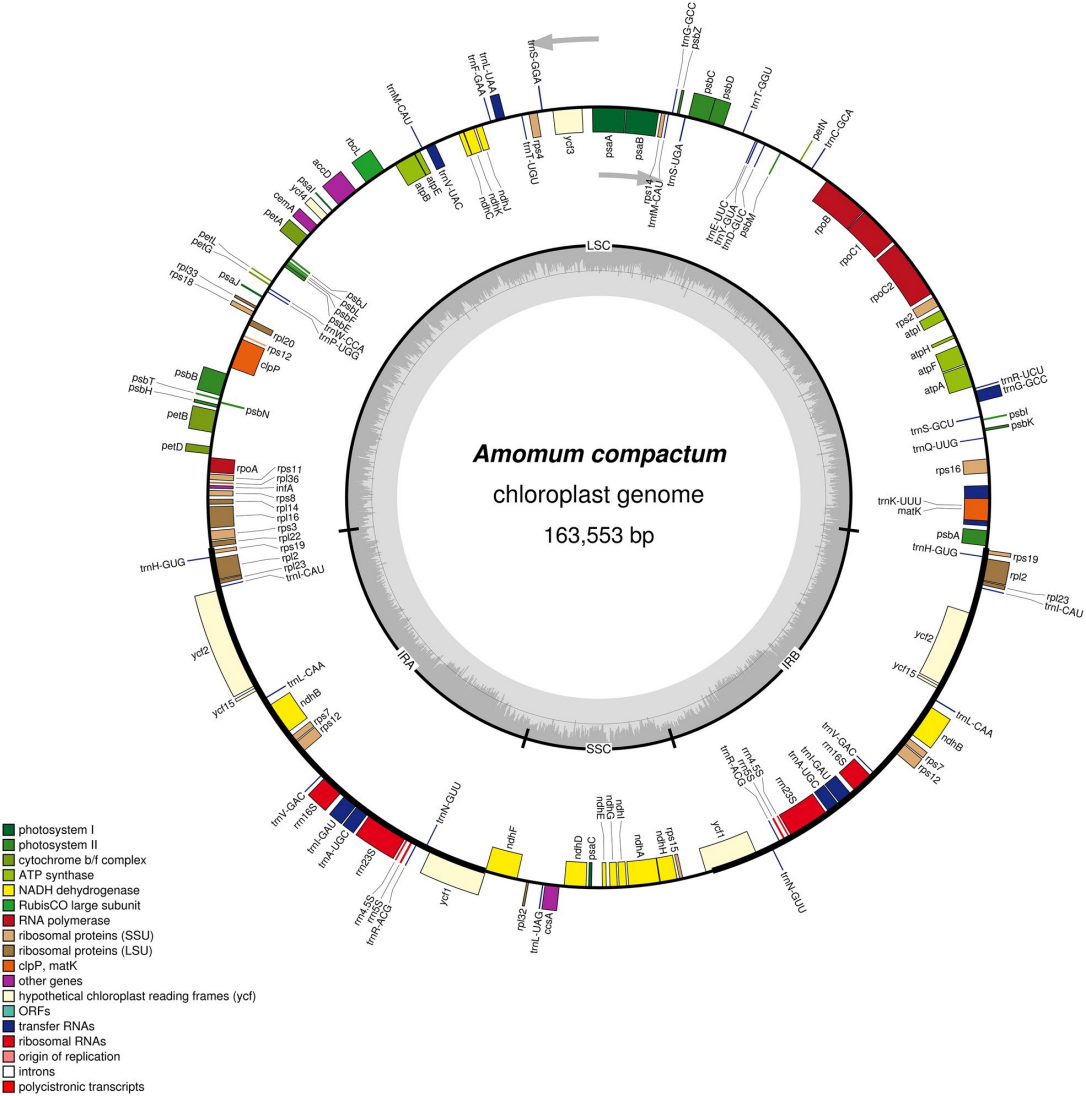
*A. compactum* cp genome map. Genes drawn outside the circle are counterclockwise, whereas inside are transcribed clockwise. Genes are color-coded according to different functional groups. The darker gray represents GC content in the inner circle, conversely the lighter one represents AT content

The complete cp genome sequence of *A. compactum* is 163,553 bp in length with a obvious quadripartite structure (Fig. 1). A pair of inverted region (IR) with 29,824 bp in length partition the rest sequence into a LSC region (88,535 bp) and a SSC region (15,370 bp) (Table 1). The universal GC content of this cp sequence was 36.0%, which has been reported to act a significant role in evolution of genomic structures. Nevertheless, the overall GC content is unequally distributed across the cp genome, which is lowest in SSC region (29.8%) but highest in IR regions (41.1%), followed by LSC region (33.7%).

**Table 1:** Base composition in the *A. compactum* cp genome

	T(U)%	C%	A%	G%	Length (bp)
LSC	33.8	17.2	32.5	16.5	88,535
IR	28.8	19.8	30.1	21.3	29,824
SSC	34.3	15.6	35.9	14.2	15,370
Total	32.3	18.3	31.7	17.8	163,553
CDS	31.6	17.2	31.5	19.8	79,701
1st position	24	18.2	31.3	26.7	26,567
2nd position	32	20.2	30.0	17.4	26,567
3rd position	39	13.1	33.1	15.3	26,567

*CDS* protein-coding regions

As shown in Fig. 1, the *A. compactum* cp genome totally encodes 135 functional genes, that 113 are unique, containing eighty protein-coding genes, twenty-nine tRNAs and four rRNAs (Table 2). Among the functional genes, all rRNAs, eight tRNAs and seven protein-coding genes are duplicated in IR regions. The LSC region includes 60 protein-coding genes and 21 tRNAs, whereas the SSC region includes 11 protein-coding genes and one tRNA gene. Among the protein-coding genes, 72 are single-copy, whereas eight are duplicated. Among the tRNA genes, 20 are single-copy genes and nine are duplicated. Among the 113 unique genes, 13 include one intron (eight protein-coding and five tRNAs) and three (*ycf3*, *clpP*, and *rps12*) include two introns (Table 2). Unusually, the *rps12* gene is trans-spliced, of which the 5′ end is situated in LSC region whereas two replicative 3′ ends are located in IRa and IRb regions respectively. What’s more, the *ndhA* gene contains the longest intron region (1033 bp).

**Table 2:** Gene content of the *A. compactum* cp genome

Gene category	Gene group	Gene name
Self-replication	rRNA genes	*rrn16*^c^, *rrn23*^c^, *rrn5*^c^, *rrn4.5*^c^
	tRNA genes	*trnH*-*GUG*^c^, *trnK*-*UUU*^a^, *trnQ*-*UUG*, *trnS*-*GCU*, *trnC*-*GCA*, *trnD*-*GUC*, *trnY*-*GUA*, *trnE*-*UUC*, *trnR*-*UCU*, *trnT*-*GGU*, *trnS*-*UGA*, *trnG*-*GCC*^c^, *trnfM*-*CAU*, *trnS*-*GGA*, *trnT*-*UGU*, *trnL*-*UAA*^a^, *trnF*-*GAA*, *trnV*-*UAC*^a^, *trnW*-*CCA*, *trnP*-*UGG*, *trnI*-*CAU*^c^, *trnL*-*CAA*^c^, *trnV*-*GAC*^c^, *trnI*-*GAU*^a, c^, *trnA*-*UGC*^a, c^, *trnR*-*ACG*^c^, *trnN*-*GUU*^c^, *trnL*-*UAG*, *trnM*-*CAU*
	Small subunit of ribosome	*rps4*, *rps14*, *rps18*, *rps2*, *rps12*^b, c^, *rps11*, *rps8*, *rps3*, *rps19*, *rps7*^c^, *rps15*, *rps16*^a^
	Large subunit of ribosome	*rpl33*, *rpl20*, *rpl36*, *rpl14*, *rpl16*^a^, *rpl22*, *rpl2*^a, c^, *rpl23*^c^, *rpl32*
	DNA dependent RNA polymerase	*rpoB*, *rpoC1*^a^, *rpoC2*, *rpoA*
	Translational initiation factor	*infA*
Genes for photosynthesis	Subunits of NADH dehydrogenase	*ndhA*^a^, *ndhB*^a, c^, *ndhC*, *ndhD*, *ndhE*, *ndhF*, *ndhG*, *ndhH*, *ndhI*, *ndhJ*, *ndhK*
	Subunits of photosystem I	*psaA*, *psaB*, *psaC*, *psaI*, *psaJ*, *ycf3*^b^, *ycf4*
	Subunits of photosystem II	*psbA*, *psbB*, *psbC*, *psbD*, *psbE*, *psbF*, *psbH*, *psbI*, *psbJ*, *psbK*, *psbL*, *psbM*, *psbN*, *psbT*, *psbZ*
	Subunits of cytochrome b/f complex	*petN*, *petA*, *petL*, *petG*, *petB*^a^, *petD*
	Subunits of ATP synthase	*atpI*, *atpH*, *atpF*^a^, *atpA*, *atpE*, *atpB*
	Large subunit of rubisco	*rbcL*
Genes of unknown function	Open reading frames (ORF, ycf)	*ycf1*, *ycf15*^c^, *ycf2*^c^
	Pseudogenes	*ycf1*

^a^Gene with one intron

^b^Gene with two introns

^c^Gene with two copies

The protein-coding gene sequences are 79,701 bp in length, which comprise 26,567 codons. And the usage frequency of codon was counted and exhibited in Table 3. In protein-coding sequences (CDSs), the AT content are 55.3% at the first codon positions, 62.0% at the second codon positions and 72.1% at the third codon positions, respectively (Table 1). Most protein-coding genes in land plant cp genomes use the standard ATG as the initiation codon. However, in the *A. compactum* cp genome, two genes use alternatives to ATG as start codon, as following: ATC for *ndhD* and ATA for *rpl2*. Relative synonymous codon usage (RSCU) is a statistics of uneven usage of synonymous and nonsynonymous codons in the coding sequences. An RSCU value < 1.00 indicates that the use of a codon is less frequent than expected, whereas a codon used more frequently will attain an RSCU value > 1.00. A total of 96.7% (29/30) of preferred synonymous codons, i.e., RSCU values > 1, end with A/U, whereas 90.6% (29/32) of non-preferred synonymous codons, i.e., RSCU values < 1, end with G/C. This codon usage pattern is similar with other reported cp genomes [21, 22], which might be driven by the high proportion of A/T. The usage of the start codon (ATG) and UGG (coding TRP) show no bias (RSCU value = 1).

**Table 3:** Codon-anticodon recognition patterns and codon usage in the *A. compactum* cp genome

Amino acid	Codon	No.	RSCU	tRNA	Amino acid	Codon	Count	RSCU	tRNA
Phe	UUU	971	1.31		Tyr	UAU	811	1.57	
Phe	UUC	516	0.69	*trnF*-*GAA*	Tyr	UAC	221	0.43	*trnY*-*GUA*
Leu	UUA	892	1.96	*trnL*-*UAA*	Stop	UAA	48	1.66	
Leu	UUG	559	1.23	*trnL*-*CAA*	Stop	UAG	22	0.76	
Leu	CUU	567	1.25		His	CAU	519	1.6	
Leu	CUC	181	0.4		His	CAC	129	0.4	*trnH*-*GUG*
Leu	CUA	381	0.84	*trnL*-*UAG*	Gln	CAA	706	1.54	*trnQ*-*UUG*
Leu	CUG	151	0.33		Gln	CAG	210	0.46	
Ile	AUU	1146	1.47		Asn	AAU	989	1.55	
Ile	AUC	426	0.55	*trnI*-*GAU*	Asn	AAC	289	0.45	*trnN*-*GUU*
Ile	AUA	763	0.98	*trnI*-*CAU*	Lys	AAA	1114	1.49	*trnK*-*UUU*
Met	AUG	614	1	*trn(f)M*-*CAU*	Lys	AAG	383	0.51	
Val	GUU	521	1.45		Asp	GAU	875	1.64	
Val	GUC	159	0.44	*trnV*-*GAC*	Asp	GAC	192	0.36	*trnD*-*GUC*
Val	GUA	559	1.56	*trnV*-*UAC*	Glu	GAA	1125	1.53	*trnE*-*UUC*
Val	GUG	194	0.54		Glu	GAG	350	0.47	
Ser	UCU	598	1.74		Cys	UGU	232	1.56	
Ser	UCC	337	0.98	*trnS*-*GGA*	Cys	UGC	66	0.44	*trnC*-*GCA*
Ser	UCA	412	1.2	*trnS*-*UGA*	Stop	UGA	17	0.59	
Ser	UCG	182	0.53		Trp	UGG	452	1	*trnW*-*CCA*
Pro	CCU	442	1.62		Arg	CGU	365	1.37	*trnR*-*ACG*
Pro	CCC	202	0.74		Arg	CGC	86	0.32	
Pro	CCA	325	1.19	*trnP*-*UGG*	Arg	CGA	342	1.29	
Pro	CCG	120	0.44		Arg	CGG	113	0.43	
Thr	ACU	537	1.57		Arg	AGA	519	1.95	*trnR*-*UCU*
Thr	ACC	237	0.7	*trnT*-*GGU*	Arg	AGG	168	0.63	
Thr	ACA	433	1.27	*trnT*-*UGU*	Ser	AGU	430	1.25	
Thr	ACG	157	0.46		Ser	AGC	102	0.3	*trnS*-*GCU*
Ala	GCU	626	1.82		Gly	GGU	604	1.39	
Ala	GCC	203	0.59		Gly	GGC	141	0.33	*trnG*-*GCC*
Ala	GCA	434	1.26	*trnA*-*UGC*	Gly	GGA	714	1.65	
Ala	GCG	112	0.33		Gly	GGG	276	0.64	

*RSCU* relative synonymous codon usage

### Repeat and SSR analysis

SSRs are a class of tandemly repeated sequences that consists of 1–6 nucleotide repeat units. SSRs are important in plant typing and widely developed as molecular genetic markers for species identification. Total 58 SSRs loci were found in the *A. compactum* cp genome (Table 4), and 47 SSRs were only composed of A/T bases. Furthermore, 10 SSRs were composed of di-nucleotide (AT/TA) repeats, and one SSR was composed of trinucleotide (ATA) repeats. Obviously, the SSRs in the *A. compactum* cp genome were rich in A/T, which has been reported in many plant families [23–25]. Among these SSRs, 17 SSRs were situated in protein-coding genes and one was located in a tRNA gene. Furthermore, five were in coding regions and 12 in intronic regions. No tetra-, penta- or hexa-nucleotide repeats over 15 bp long was detected. REPuter allowed us to identify 24 repeats, including 13 forward and 11 palindromic repeats (Table 5). Almost all repeats were situated in the intronic and intergenic regions, although few of them were situated in protein-coding regions [26]. As reported in other genomes, the gene richest in repeats was *ycf2*, carrying two direct and two palindromic repeats.

**Table 4:** Simple sequence repeats in the *A. compactum* cp genome

cpSSR ID	Repeat motif	Length (bp)	Start	End	Region	Annotation
1	(T)10	10	3975	3984	LSC	*trnK*-*UUU*
2	(A)10	10	4328	4337	LSC	
3	(TA)6	12	4900	4911	LSC	
4	(A)10	10	5287	5296	LSC	*rps16* intron
5	(A)11	11	6253	6263	LSC	
6	(TA)6	12	6609	6620	LSC	
7	(A)10	10	7204	7213	LSC	
8	(AT)6	12	7521	7532	LSC	
9	(A)10	10	7700	7709	LSC	
10	(T)12	12	8633	8644	LSC	
11	(A)13	13	14,885	14,897	LSC	
12	(T)10	10	17,474	17,483	LSC	
13	(A)10	10	19,831	19,840	LSC	*rpoC2*
14	(T)11	11	24,121	24,131	LSC	*rpoC1* intron
15	(A)10	10	28,802	28,811	LSC	
16	(A)15	15	29,013	29,027	LSC	
17	(A)11	11	30,868	30,878	LSC	
18	(T)10	10	35,129	35,138	LSC	
19	(TA)7	14	38,632	38,645	LSC	
20	(A)12	12	39,292	39,303	LSC	
21	(A)12	12	47,481	47,492	LSC	
22	(T)10	10	48,986	48,995	LSC	
23	(A)10	10	50,236	50,245	LSC	
24	(AT)7	14	50,395	50,408	LSC	
25	(T)10	10	51,829	51,838	LSC	
26	(T)11	11	52,709	52,719	LSC	
27	(ATA)5	15	54,345	54,359	LSC	
28	(A)11	11	54,562	54,572	LSC	
29	(T)10	10	58,778	58,787	LSC	
30	(T)11	11	59,269	59,279	LSC	
31	(A)12	12	60,919	60,930	LSC	
32	(T)10	10	61,621	61,630	LSC	
33	(AT)6	12	63,489	63,500	LSC	
34	(A)12	12	68,715	68,726	LSC	
35	(AT)10	20	69,266	69,285	LSC	
36	(T)10	10	70,716	70,725	LSC	
37	(A)10	10	72,600	72,609	LSC	*rps18*
38	(TA)7	14	74,094	74,107	LSC	*rps12* intron
39	(A)10	10	74,569	74,578	LSC	*clpP* intron
40	(T)11	11	74,845	74,855	LSC	*clpP* intron
41	(T)10	10	75,108	75,117	LSC	*clpP* intron
42	(T)10	10	75,572	75,581	LSC	*clpP* intron
43	(T)10	10	75,831	75,840	LSC	*clpP* intron
44	(A)10	10	79,177	79,186	LSC	
45	(AT)6	12	79,751	79,762	LSC	*petB* intron
46	(T)10	10	86,407	86,416	LSC	*rpl16* intron
47	(T)11	11	88,970	88,980	IRa	
48	(T)10	10	116,573	116,582	IRa	*ycf1*
49	(A)11	11	120,872	120,882	SSC	
50	(T)11	11	121,055	121,065	SSC	
51	(A)11	11	128,865	128,875	SSC	*ndhA* intron
52	(T)10	10	129,188	129,197	SSC	*ndhA* intron
53	(AT)6	12	131,778	131,789	SSC	
54	(T)11	11	133,103	133,113	SSC	
55	(T)12	12	133,236	133,247	SSC	
56	(T)11	11	133,374	133,384	SSC	*ycf1*
57	(A)10	10	135,507	135,516	IRb	*ycf1*
58	(A)11	11	163,109	163,119	IRb

**Table 5:** Long repeat sequences in *A. compactum* cp genome

ID	Repeat start 1	Type	Size (bp)	Repeat start 2	Mismatch (bp)	E value	Gene	Region
1	3990	P	34	3996	− 3	4.12E−06	*trnK*-*UUU* (intron)	LSC
2	8768	P	31	48,057	− 3	1.98E−04	IGS; *trnS*-*GGA*	LSC
3	10,522	F	30	39,347	− 3	7.15E−04	*trnG*-*GCC* (intron)	LSC
4	31,322	P	32	31,352	− 3	5.46E−05	IGS	LSC
5	32,991	F	30	33,020	− 3	7.15E−04	IGS	LSC
6	39,660	P	32	39,701	0	4.08E−10	IGS	LSC
7	41,551	F	58	43,775	− 3	7.54E−20	*psaB*; *psaA*	LSC
8	41,595	F	37	43,819	− 2	2.39E−09	*psaB*; *psaA*	LSC
9	63,481	P	31	126,101	− 3	1.98E−04	IGS	LSC; SSC
10	63,481	F	31	126,106	− 3	1.98E−04	IGS	LSC; SSC
11	63,487	F	32	69,264	− 3	5.46E−05	IGS	LSC
12	67,809	P	31	67,864	− 2	6.83E−06	IGS	LSC
13	71,632	F	30	71,659	0	6.53E−09	IGS	LSC
14	72,281	F	42	72,302	− 3	1.21E−10	*rps18*	LSC
15	91,249	F	46	91,299	− 1	2.10E−16	*trnI*-*CAU*; IGS	IRa
16	91,249	P	46	160,743	− 1	2.10E−16	*trnI*-*CAU*; IGS	IRa; IRb
17	91,299	P	46	160,793	− 1	2.10E−16	IGS	IRa; IRb
18	93,917	F	30	93,938	− 3	7.15E−04	*ycf2*	IRa
19	93,917	P	30	158,120	− 3	7.15E−04	*ycf2*	IRa; IRb
20	93,938	P	30	158,141	− 3	7.15E−04	*ycf2*	IRa; IRb
21	121,695	P	30	121,723	− 3	7.15E−04	IGS	SSC
22	158,122	F	30	158,143	− 3	7.15E−04	*ycf2*	IRb
23	160,743	F	46	160,793	− 1	2.10E−16	IGS	IRb
24	160,762	F	30	160,812	− 3	7.15E−04	IGS	IRb

*F* forward, *P* palindromic, *IGS* intergenic space

### IR expansion/contraction in the *A. compactum* cp genome

**Fig. 2:**
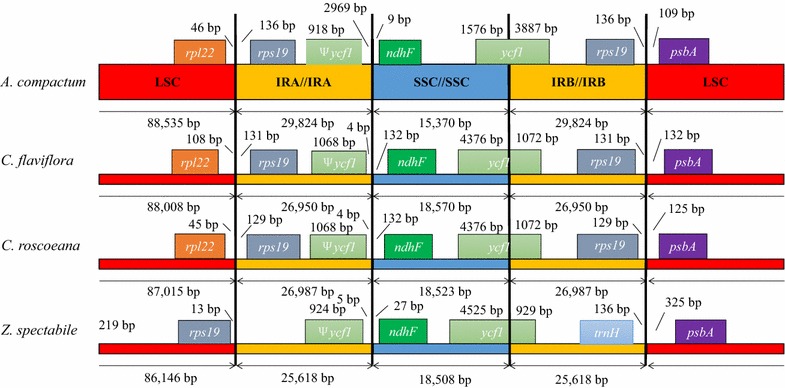
Comparison of the border positions of the LSC, SSC, and IR regions among four complete Zingiberaceae chloroplast genomes. Gene names are indicated in boxes, and their lengths in the corresponding regions are displayed above the boxes

The variations of angiosperm cp genomes in length are mainly because of the contraction and expansion of boundary regions between the IR regions with single copy (SC) regions. A minute comparison of junctional regions between the IR and SC boundaries among *A. compactum*, *C. flaviflora*, *C. roscoeana*, and *Z. spectabile* is presented in Fig. 2. In addition, a size comparison of cp genome among the four Zingiberaceae species is shown in Additional file 2: Table S1. In spite of the alike lengths of IR regions in these four species (from 25,618 to 29,824 bp), few IR contractions/expansions were still detected. *rpl22*, *ycf1* and *rps19* pseudogenes with various lengths were situated in IRb/LSC or IRb/SSC boundaries. The borderline of the IRb/LSC junction was situated in left side of the *rps19* gene in examined cp genomes, except in *Z. spectabile*, which resulted from the contraction of the IRa region in the *Z. spectabile* cp genome. By contrast, the *ycf1* pseudogene was situated in the left side of the IRa-SSC border and was 4–5 bp away from the IRa-SSC borderline, except in the *A. compactum* cp genome. The size of the *ycf1* pseudogene was 918 bp in *A. compactum*, 1068 bp in *C. flaviflora* and *C. roscoeana*, and 924 bp in *Z. spectabile*. In addition, in the *A. compactum* cp genome, the *ycf1* pseudogene was 2969 bp away from the IRa-SSC borderline, that indicated the expansion of the IR region. The *trnH* gene was situated in LSC region, except in *Z. spectabile* cp genome, where it was situated in SSC region and was 136 bp away from the IRb-LSC borderline.

### Comparison with other Zingiberaceae cp genomes

**Fig. 3:**
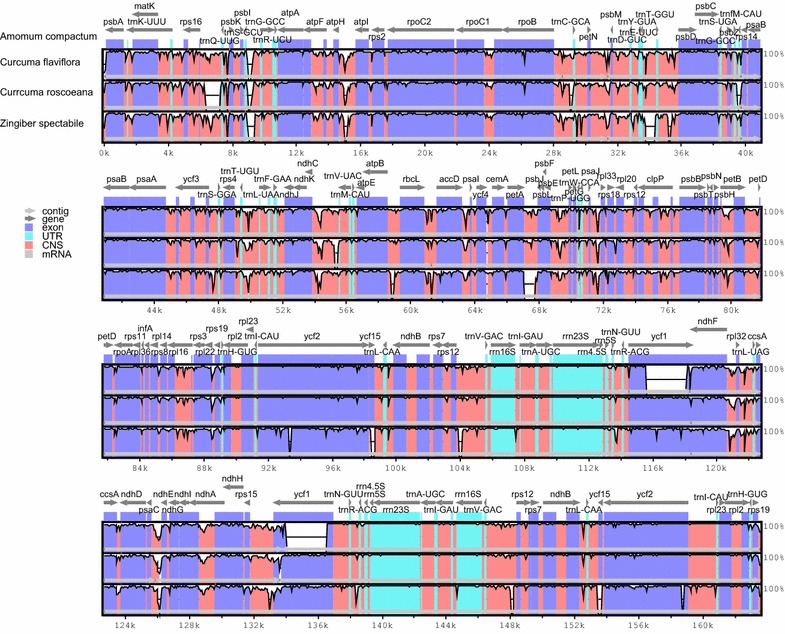
Sequence comparison of the *A. compactum*, *C. flaviflora*, *C. roscoeana* and *Z. spectabile* cp genomes generated by mVISTA. Black lines designate regions of sequence identity by a 50% identity cutoff with *A. compactum*. Dashed rectangles indicate highly divergent regions of *A. compactum* compared with *C. flaviflora*, *C. roscoeana* and *Z. spectabile*

Three sequences representing the Zingiberaceae (*C. flaviflora*, *C. roscoeana* and *Z. spectabile*) were selected for comparison with *A. compactum*. Pairwise cp genome alignments between *A. compactum* and other three cp genomes regained a high degree of synteny (Additional file 3: Figure S1, Additional file 4: Figure S1 and Additional file 5: Figure S3). To detect the divergent regions in the cp genome, this study compared the sequence identities among four Zingiberaceae cp genomes by mVISTA, using the annotation of *A. compactum* as a reference. The multiple sequences alignment showed the coding regions are highly conserved, however the non-coding regions are divergent (Fig. 3). As an example, the intergenic sequences between the *trnT*-*GGU*–*psbD*, *rps16*–*trnQ*-*UUG*, *atpH*–*atpI*, *trnE*-*UUC*–*trnT*-*GGU*, *trnT*-*UGU*–*trnL*-*UAA*, *petA*–*psbL* and *psaC*–*ndhE* regions were highly divergent, parts of which have been also reported as divergent sequences in other plant. Obviously, the LSC region and SSC region were more divergent than IR regions.

### Phylogenetic analysis

**Fig. 4:**
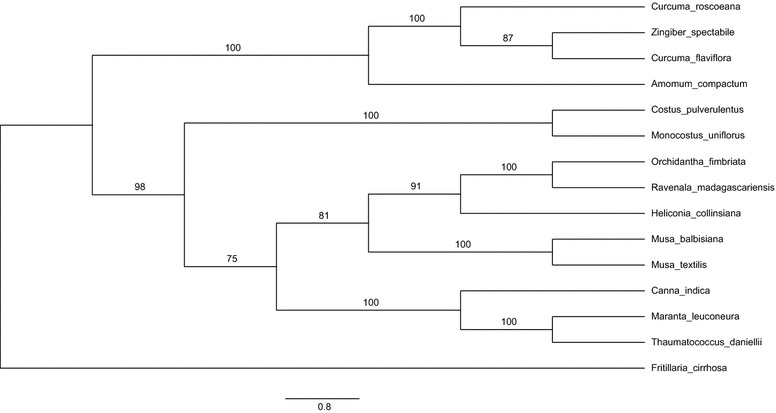
MP phylogenetical tree of 15 Zingiberales species, on basis of 67 protein-coding gene sequences in the cp genomes. Bootstrap values are indicated upon the branches

**Fig. 5:**
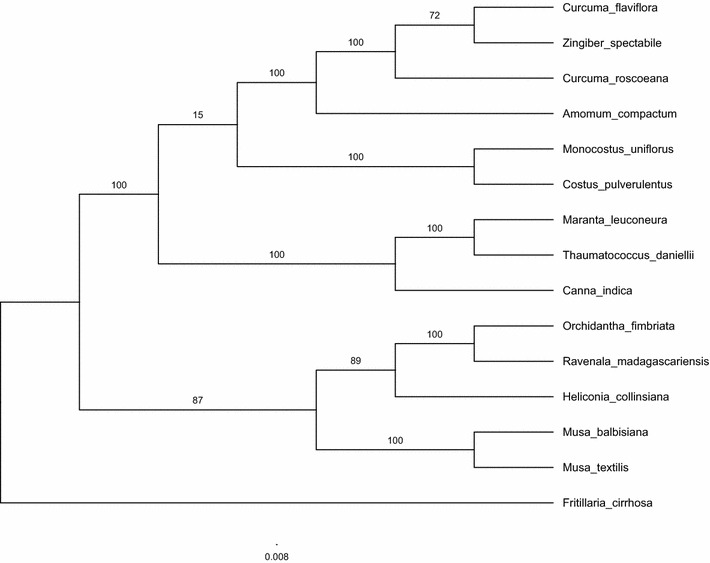
ML phylogenetical tree of 15 Zingiberales species, on basis of 67 protein-coding gene sequences in the cp genome. Bootstrap values are indicated upon the branches

Cp genomes are widely employed in the study of evolution through phylogenetics. To examine the phylogenetic position of *A. compactum* and its relationship within Zingiberales, MP and ML phylogenetical analyses were performed based on 67 protein-coding gene sequences from 15 plant taxa, including *A. compactum*, as sequenced in the study. The total alignment was 51,452 bp in length. The results are presented in Figs. 4 and 5. The basic topologies were similar in the MP and ML analyses, but there were few differences. Bootstrap values were all extremely high, and nine of the 12 nodes with bootstrap values of ≥ 90% were found in MP tree, whereas eight of 12 nodes were found in ML tree with 100% bootstrap values. The Zingiberaceae species *A. compactum*, *C. flaviflora*, *C. roscoeana and Z. spectabile* were grouped in both MP and ML phylogenetic trees with 100% bootstrap values. In the MP trees, the four Zingiberaceae species composed a unique clade and were separated from the rest of Zingiberales with high bootstrap values in every node. By contrast, the ML tree was mainly separated into two clades, one of which included Strelitziaceae, Heliconiaceae, Musaceae and Lowiaceae species, whereas another included Zingiberaceae, Costaceae, Cannaceae and Marantaceae species. However, the Zingiberaceae and Costaceae species were grouped with a very low bootstrap value (15%) in the ML tree. These phylogenetic results strongly support the position of *A. compactum* and provide some helpful hints about relationships within the order Zingiberales.

## Conclusion

The research assembled, annotated and analyzed the whole cp genome of *A. compactum*, which reveals that the cp genome of *A. compactum* shares a quadruple structure, gene order, GC content, and codon usage features, similar to those of other land plant cp genomes. This *Amomum* cp genome was compared with three available Zingiberaceae cp genomes, while the genome structure and composition are similar. Also phylogenetic analysis provides new insight into phyletic evolution of this genus. Our research will contribute to species identification and evolutionary mechanisms required for the further study of *A. compactum.*

## Additional files

**Additional file 1.** Minimum Standards of Reporting Checklist.

**Additional file 2: Table S1.** Size comparison of *A. compactum* cp genomic regions with those of 3 other Zingiberaceae cp genomes.

**Additional file 3: Figure S1.** Chloroplast genomic alignment between *A. compactum* and *C. flaviflora* (KR967361).

**Additional file 4: Figure S2.** Chloroplast genomic alignment between *A. compactum* and *C. roscoeana* (KF601574).

**Additional file 5: Figure S3.** Chloroplast genomic alignment between *A. compactum* and *Z. spectabile* (JX088661).

## Abbreviations

cp: chloroplast
LSC: large single copy
SSC: small single copy
IR: inverted repeat
tRNA: transfer RNA
rRNA: ribosomal RNA
