# Supplementary material for: Complete chloroplast genome of the medicinal plant Amomum compactum: gene organization, comparative analysis, and phylogenetic relationships within Zingiberales
Source: Chin Med. 2018 Feb 13;13:10. doi: 10.1186/s13020-018-0164-2 (PMC5811967; doi:10.1186/s13020-018-0164-2)
Supplement: Supplementary file 2 — Additional file 2: Table S1. Size comparison of A. compactum cp genomic regions with those of 3 other Zingiberaceae cp genomes. [file 13020_2018_164_MOESM2_ESM.docx]

**Additional file 2: Table S1** Size comparison of *A. compactum* cp genomic regions with those of 3 other Zingiberaceae cp genomes.

| **Species** | **Length (bp)** | | | |
| --- | --- | --- | --- | --- |
|  | **Total genome LSC SSC IR** | | | |
| *Amomum compactum* | 163,553 | 88,535 | 15,370 | 29,824 |
| *Curcuma flaviflora* | 160,478 | 88,008 | 18,570 | 26,950 |
| *Curcuma roscoeana* | 159,512 | 87,015 | 18,523 | 26,987 |
| *Zingiber spectabile* | 155,890 | 86,146 | 18,508 | 25,618 |
