# Supplementary figures and images for: Complete chloroplast genome of the medicinal plant Amomum compactum: gene organization, comparative analysis, and phylogenetic relationships within Zingiberales
Source: Chin Med. 2018 Feb 13;13:10. doi: 10.1186/s13020-018-0164-2 (PMC5811967; doi:10.1186/s13020-018-0164-2)

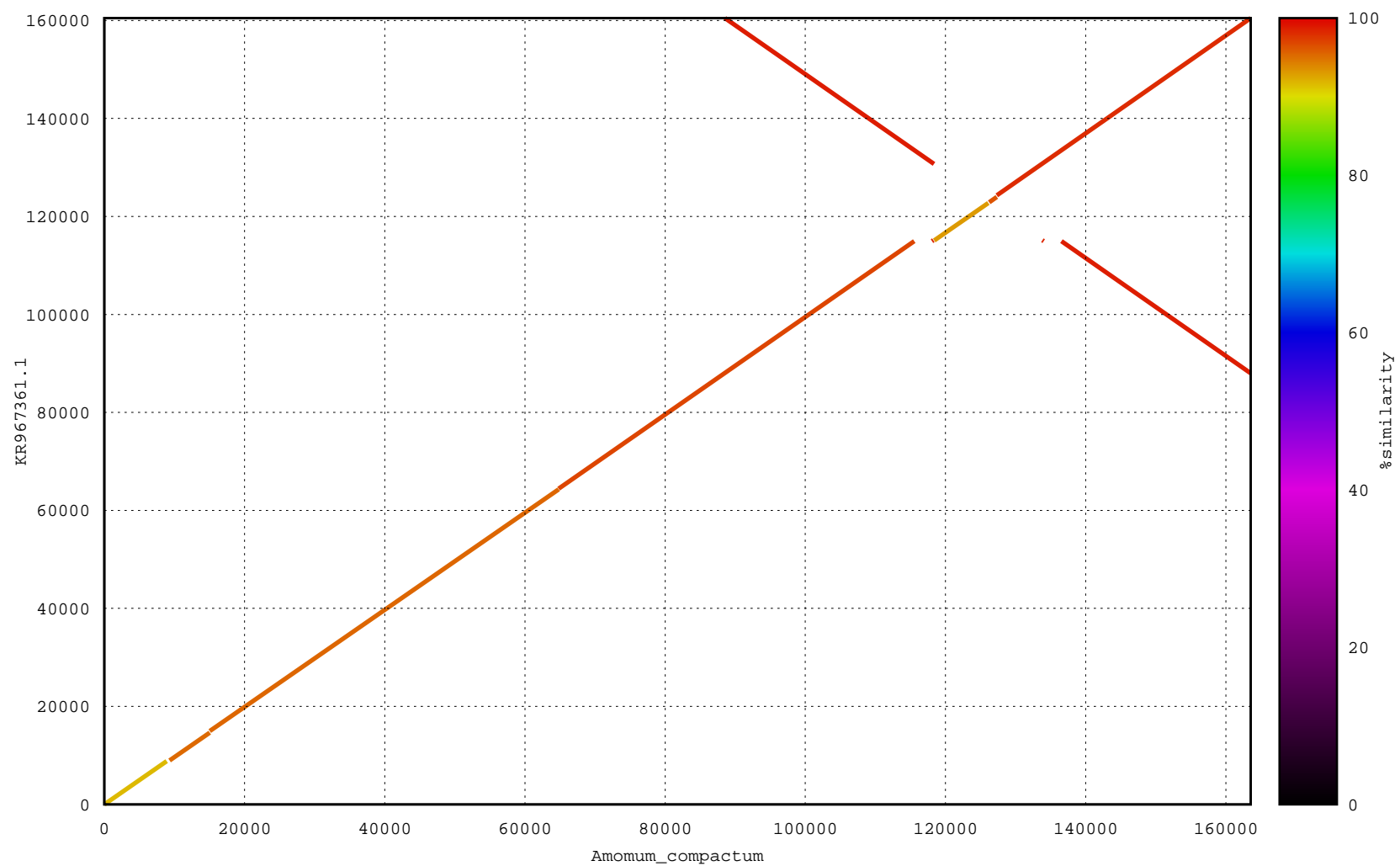

Supplement: Supplementary file 3 — Additional file 3: Figure S1. Chloroplast genomic alignment between A. compactum and C. flaviflora (KR967361). [file 13020_2018_164_MOESM3_ESM.pdf]

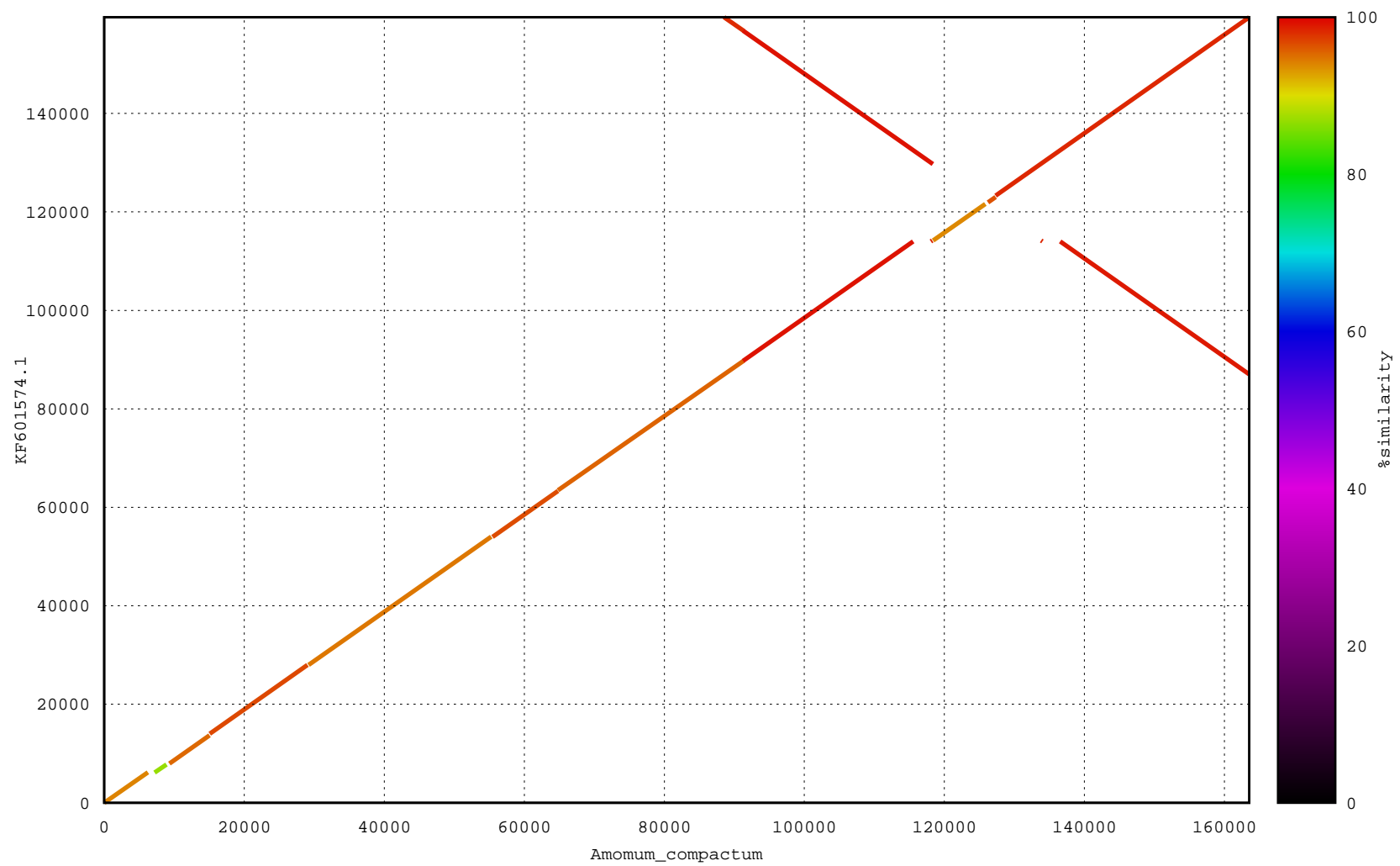

Supplement: Supplementary file 4 — Additional file 4: Figure S2. Chloroplast genomic alignment between A. compactum and C. roscoeana (KF601574). [file 13020_2018_164_MOESM4_ESM.pdf]

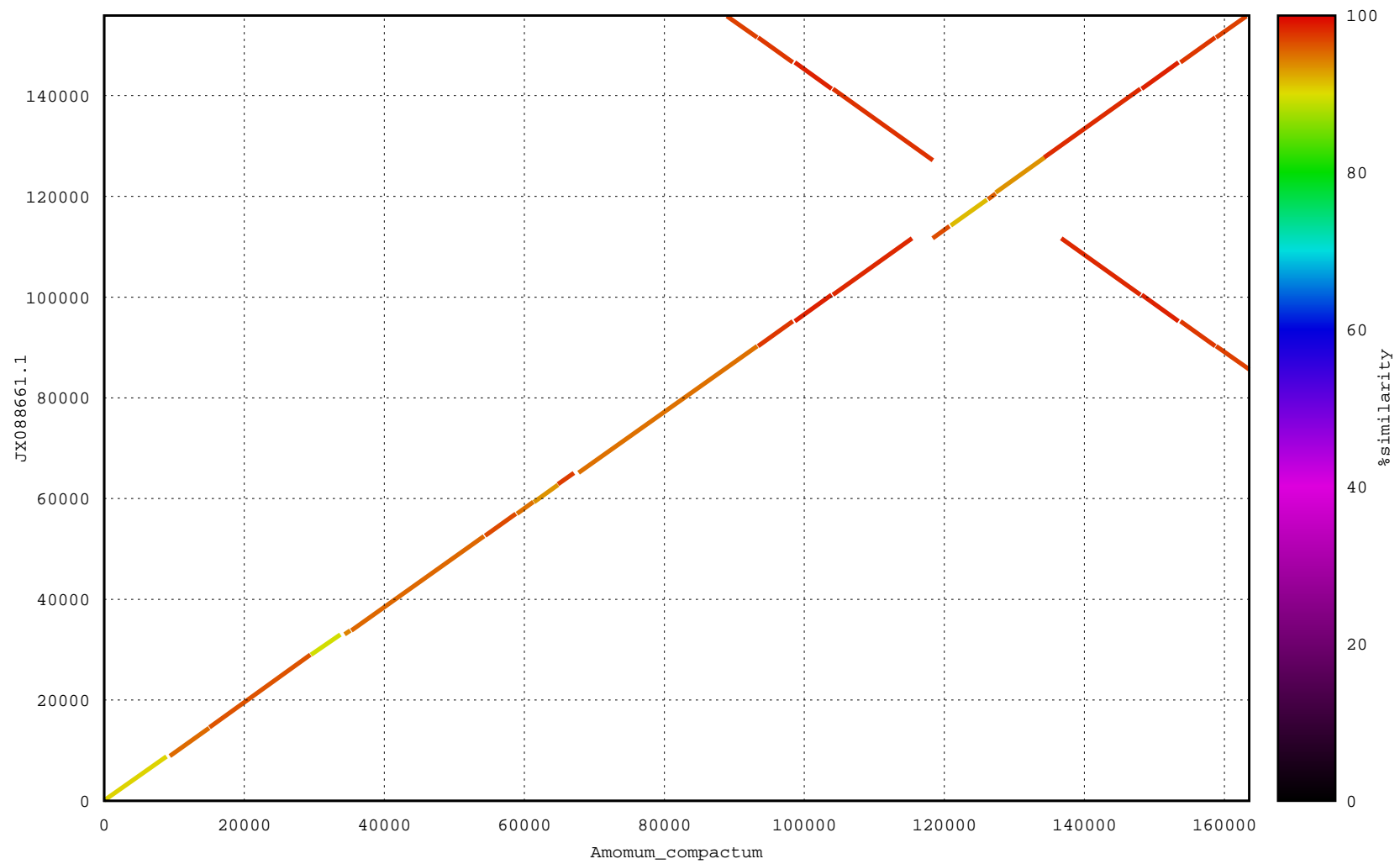

Supplement: Supplementary file 5 — Additional file 5: Figure S3. Chloroplast genomic alignment between A. compactum and Z. spectabile (JX088661). [file 13020_2018_164_MOESM5_ESM.pdf]
